# Swine Influenza Virus Induces RIPK1/DRP1-Mediated Interleukin-1 Beta Production

**DOI:** 10.3390/v10080419

**Published:** 2018-08-09

**Authors:** Hong-Su Park, Guanqun Liu, Qiang Liu, Yan Zhou

**Affiliations:** 1 Vaccine and Infectious Disease Organization—International Vaccine Centre (VIDO-InterVac), University of Saskatchewan, Saskatoon, SK S7N 5E3, Canada; hongsu.park@usask.ca (H.-S.P.); guanqun.liu@usask.ca (G.L.); qiang.liu@usask.ca (Q.L.); 2 Department of Veterinary Microbiology, Western College of Veterinary Medicine, University of Saskatchewan, Saskatoon, SK S7N 5B4, Canada; 3 Vaccinology and Immunotherapeutics Program, School of Public Health, University of Saskatchewan, Saskatoon, SK S7N 2Z4, Canada

**Keywords:** swine influenza virus, interleukin-1 beta, NLRP3 inflammasome, receptor-interacting protein kinase 1, dynamin-related protein 1, mitochondrial fission, porcine alveolar macrophage

## Abstract

Nucleotide-binding domain and leucine-rich repeat-containing protein 3 (NLRP3) inflammasome plays a pivotal role in modulating lung inflammation in response to the influenza A virus infection. We previously showed that the swine influenza virus (SIV) infection induced NLRP3 inflammasome-mediated IL-1β production in primary porcine alveolar macrophages (PAMs), and we were interested in examining the upstream signaling events that are involved in this process. Here, we report that the SIV-infection led to dynamin-related protein 1 (DRP1) phosphorylation at serine 579 and mitochondrial fission in PAMs. IL-1β production was dependent on the reactive oxygen species (ROS) production, and DRP1 phosphorylation resulted in the upregulation of the NLRP3 inflammasome. Furthermore, the requirement of the kinase activity of receptor-interacting protein kinase 1 (RIPK1) for the IL-1β production and RIPK1-DRP1 association suggested that RIPK1 is an upstream kinase for DRP1 phosphorylation. Our results reveal a critical role of the RIPK1/DRP1 signaling axis, whose activation leads to mitochondrial fission and ROS release, in modulating porcine NLRP3 inflammasome-mediated IL-1β production in SIV-infected PAMs.

## 1. Introduction

Interleukin-1 beta (IL-1β) is a pro-inflammatory cytokine that contributes to the effective modulation of the host innate and adaptive immunity upon influenza A virus (IAV) infection [1,2,3]. IL-1β is tightly regulated by pattern recognition receptors (PRRs) such as nucleotide-binding domain and leucine-rich repeat-containing protein 3 (NLRP3), which functions by forming the NLRP3 inflammasome with its adaptor protein, apoptosis-associated speck-like protein containing the caspase recruitment domain (ASC), and procaspase-1. The activation of NLRP3 leads to the conversion of procaspase-1 into caspase-1, which cleaves pro-IL-1β to generate mature IL-1β and also induces pyroptosis [4,5].

Mitochondria are well appreciated for their roles in innate immunity. Mitochondrial proteins are involved in the NLRP3 inflammasome regulation [6,7,8]. Pathophysiological changes mediated by mitochondria including the imbalance in mitochondrial dynamics and the release of cytochrome c, mitochondrial DNA or reactive oxygen species (ROS) can either activate or inhibit the NLRP3 inflammasome [9,10,11,12,13]. The phosphorylation of dynamin-related protein 1 (DRP1), a guanosine triphosphate (GTP) hydrolase (GTPase), on different residues determines the mitochondrial dynamics, namely fission and fusion. For human DRP1 (transcript variant 1), phosphorylation at serine 616 (S616) or dephosphorylation at serine 637 (S637) is linked to mitochondrial fission, whereas phosphorylation at S637 is associated with fusion [14]. In order for fission to occur, DRP1 is recruited to mitochondria upon its phosphorylation at S616 by kinases including receptor-interacting protein kinase 1 (RIPK1) or dephosphorylation at S637 [15,16,17,18]. The association of DRP1 with mitochondrial fission proteins leads to mitochondrial fission, which depends on GTP hydrolysis by DRP1 [19]. The suppression of mitochondrial fission by diverse molecules leads to the inhibition of NLRP3 inflammasome activity [20,21,22], while this is contradicted by a finding that the impaired mitochondrial fission can upregulate the NLRP3 inflammasome activity [12].

Viruses can alter the mitochondrial dynamics to subvert the host’s innate immunity or to modulate the host cell survival. Various RNA viruses are reported to induce DRP1-mediated mitochondrial fission to regulate apoptosis [23,24,25,26], whereas the dengue virus is reported to regulate mitochondrial fusion [27,28,29]. An initial study on DRP1-mediated NLRP3 inflammasome activation upon viral infection discovered that in response to vesicular stomatitis virus (VSV), RIPK1 forms a complex with RIPK3 to induce necroptosis, while it also phosphorylates DRP1. The latter event leads to mitochondrial fission, and subsequently, ROS can activate NLRP3 leading to IL-1β production [16]. In contrast, another report showed that the NLRP3 inflammasome activation upon VSV infection does not require RIPK1 or DRP1 [30]. In addition, there are reports showing that cyclin-dependent kinase 1 is required for DRP1 phosphorylation and mitochondrial fission upon infection with RNA viruses such as hepatitis C virus and rotavirus [23,26]. Upon IAV infection, a mitochondrial fusion protein, mitofusin 2, can regulate the NLRP3 inflammasome activation [7]. The IAV PB1-F2 protein, which translocates to mitochondria, can cause mitochondrial fission that is associated with the defective NLRP3 inflammasome activation [11]. However, there is still a lack of information on how DRP1-mediated mitochondrial fission fits in the innate immune signaling in IAV-infected cells.

Our previous study demonstrated that the swine influenza virus (SIV) infection induced NLRP3 inflammasome activation leading to the IL-1β production in primary porcine alveolar macrophages (PAMs) [31]. In the present study, we investigated whether DRP1-mediated mitochondrial dynamics are involved in this process. Our results suggest that upon SIV infection of PAMs, mitochondrial fission occurred through the RIPK1/DRP1 signaling axis, which is involved in the NLRP3 inflammasome-mediated IL-1β production.

## 2. Materials and Methods

### 2.1. Cells and Viruses

PAMs were isolated from lungs of 4-week-old, SIV-negative piglets by collecting bronchoalveolar lavage fluid, where most of the cells are comprised of alveolar macrophages [32,33]. The PAMs were characterized by flow cytometry using mouse anti-pig macrophage antibody conjugated with fluorescein isothiocyanate (MCA2317F, Bio-Rad, Mississauga, ON Canada) [31,34]. PAMs were cultured with HyClone RPMI 1640 medium (SH30027.01, GE Healthcare, Mississauga, ON, Canada) supplemented with a 20% fetal bovine serum (FBS) (16000-044, Thermo Fisher, Mississauga, ON, Canada), 50 μg/mL gentamicin (BS724, Bio Basic Canada, Markham, ON, Canada), and 1× Antibiotic-Antimycotic (15240-062, Thermo Fisher). Human embryonic kidney 293T (HEK293T) cells were maintained in Dulbecco’s modified Eagle’s medium (D5796, Sigma, Oakville, ON, Canada) supplemented with 10% FBS and 50 μg/mL gentamicin. The SIV strain, influenza A/swine/Saskatchewan/18789/2002/H1N1 (Sk02) was propagated in Madin-Darby canine kidney cells and titrated by plaque assay as previously described [31].

### 2.2. Antibodies and Reagents

Rabbit polyclonal NP and NS1 antibodies were produced in our lab [35]. The commercial antibodies used are as follows. Goat anti-porcine IL-1β antibody (BAF681): R&D Systems (Minneapolis, MN, USA); rabbit anti-porcine caspase-1 (p20) antibody (PAB592Po01): Cloud-Clone Corp. (Houston, TX, USA); mouse anti-Myc-tag antibody (#2276), mouse anti-β-actin antibody (#3700), rabbit anti-DRP1 antibody (#8570) and rabbit anti-phospho-DRP1 (S616) antibody (#3455): Cell Signaling Technology (Beverly, MA, USA); mouse anti-FLAG M2 antibody (F3165): Sigma; IRDye 680RD donkey anti-rabbit (926-68073), IRDye 800CW donkey anti-mouse (926-32212) and IRDye 800CW donkey anti-goat (926-32214) antibodies: LI-COR Biosciences (Lincoln, NE, USA). The following reagents were used: lipopolysaccharide (LPS) (L3024) and N-acetyl L-cysteine (NAC) (A9165): Sigma; Necrostatin-1 (Nec-1) (BML-AP309): Enzo Life Sciences (Farmingdale, NY USA); Mdivi-1 (ab144589): Abcam (Cambridge, MA, USA).

### 2.3. Plasmid Construction

Plasmids expressing porcine NLRP3, ASC, procaspase-1, and pro-IL-1β were generated using cDNA synthesized from the total RNA of PAMs that were stimulated with LPS [31]. Using the same cDNA, the full-length porcine DRP1 was cloned into pcDNA3.1-3×Myc (C-terminal tag) generating pcDNA-DRP1-Myc. Primers were designed based on the GenBank sequence of porcine DRP1 transcript variant X1 (accession number: XM_021092056). Myc-tagged DRP1 constructs with S579D or S579A mutation (pcDNA-DRP1 (S579D)-Myc or pcDNA-DRP1 (S579A)-Myc) were generated by site-directed mutagenesis using pcDNA-DRP1-Myc as the template. Porcine RIPK1 was cloned into pcDNA3.1-3×Myc or pCMV-3×Flag (N-terminal tag) using cDNA from porcine alveolar macrophage cell line, 3D4/2, generating pcDNA-RIPK1-Myc or pCMV-Flag-RIPK1. Using the above construct as the template, the mutant RIPK1 construct with K41A/K42A (pcDNA-RIPK1 (K41A/K42A)-Myc) was generated by mutagenesis. Myc-tagged human RIPK1 (#44159, Addgene, Cambridge, MA, USA) was a gift from Xin Lin [36]. HA/GFP-tagged human RIPK1 (K45A) (#41389, Addgene) was a gift from Francis Chan [37] and used as the template to generate Myc-tagged human RIPK1 (K45A). Primer sequences are available upon request. All the mutations were confirmed by nucleotide sequencing.

### 2.4. Infection and Treatment of PAMs

PAMs seeded at 1 × 10^6^ cells per well on 24-well plates were either infected with the Sk02 virus for 20 h at a multiplicity of infection (MOI) of 1 or stimulated with 200 ng/mL LPS for 12 h. The cells were pre-treated with vehicles or inhibitors for 2 h, and were then infected with the virus or stimulated with LPS in the presence of the vehicles or inhibitors. Nec-1 was used at 80 or 160 μM for RIPK1 inhibition; Mdivi-1 was used at 5 or 20 μM for DRP1 inhibition, while dimethyl sulfoxide (DMSO) was used as the vehicle. As a ROS scavenger, NAC was used at 2 or 10 mM and distilled water was used as the vehicle. Cell-free supernatants were collected for enzyme-linked immunosorbent assay (ELISA). Cells were lysed with 1× sodium dodecyl sulfate (SDS) sample buffer and boiled for Western blotting.

To detect the phosphorylated DRP1 (phospho-DRP1), the PAMs were seeded at 3 × 10^6^ cells per well on 6-well plates. The cells were infected with the Sk02 virus for 4 h at an MOI of 1 or stimulated with 200 ng/mL LPS for 12 h. The cells in each well were lysed with 120 μL of M-PER Mammalian Protein Extraction Reagent (78503, Thermo Fisher) containing 1× Halt Protease and Phosphatase Inhibitor Cocktail (78440, Thermo Fisher). After centrifugation, the cell lysates were mixed with 5× SDS sample buffer and were subjected to Western blotting.

### 2.5. RNA Interference and Real-Time PCR

Small-interfering RNA (siRNA) targeting porcine *RIPK1* was designed by the siRNA design tool provided by Dharmacon (Lafayette, LA, USA). The *RIPK1*-targeting siRNA (5′-UGGAAGAGGAUGUGAAGAAUU-3′) and a negative control siRNA (D-001810-01-05, 5′-UGGUUUACAUGUCGACUAA-3′) were purchased from Dharmacon. PAMs seeded at 1 × 10^6^ cells per well on 24-well plates were transfected with 100 nM of the control siRNA or RIPK1-targeting siRNA using Lipofectamine RNAiMAX (13778-030, Thermo Fisher). At 24 h post-transfection (hpt), the cells were infected with the Sk02 virus for 16 h at an MOI of 1. To show the knockdown efficiency, the relative mRNA expression of *RIPK1* compared to that of porcine hypoxanthine phosphoribosyltransferase 1 (*HPRT1*) gene was measured by real-time PCR using cDNA from total RNA with a standard protocol. Primers for porcine *RIPK1* were designed based on the GenBank sequence (accession number XM_005665536) using an online tool, Primer3 [38]. Primers for the housekeeping gene, *HPRT1* were designed based on the GenBank sequence (accession number NM_001032376). Primer sequences are available upon request.

### 2.6. NLRP3 Inflammasome Reconstitution Assay

The porcine NLRP3 inflammasome reconstitution assay was conducted to examine the effects of ectopically expressed proteins on NLRP3 inflammasome activity [31]. Briefly, the HEK293T cells seeded at 1.5 × 10^5^ cells per well on 24-well plates were co-transfected with expression plasmids for porcine NLRP3 inflammasome components and pro-IL-1β (pcDNA-NLRP3 (30 ng), pcDNA-ASC (20 ng), pCMV-Flag-procaspase-1 (20 ng), and pcDNA-pro-IL-1β (100 ng)) using *Trans*IT-LT1 Transfection Reagent (MIR2300, Mirus Bio, Madison, WI, USA). Other plasmids (50 ng of Myc-vector or Myc-tagged human RIPK1 wild-type (WT)/mutant, 100 ng of Myc-vector or Myc-tagged porcine RIPK1 WT/mutant, 100 ng of Myc-vector or Myc-tagged DRP1 WT/mutant) were co-transfected to study their effects on NLRP3 inflammasome activity. At 16 hpt, supernatants were harvested for IL-1β ELISA and the pelleted cells were lysed for Western blotting.

### 2.7. Co-Immunoprecipitation (Co-IP)

HEK293T cells were seeded at 9 × 10^5^ per well on 6-well plates and were transfected with 1 μg of Flag-tagged vector or Flag-tagged RIPK1 and Myc-tagged DRP1. At 24 hpt, the cells were lysed with 500 μL lysis buffer (50 mM Tris, pH 7.4, 150 mM NaCl, 0.5% Nonidet P-40 substitute, 1× protease inhibitor cocktail) and clarified by centrifugation at 12,000× *g* at 4 °C for 10 min. For input, 10% of the cell lysates were mixed with 5× SDS sample buffer and boiled at 95 °C for 5 min. For each sample, 35 μL of Dynabeads Protein G (10004D, Thermo Fisher) were first conjugated with 1 μg of mouse monoclonal anti-FLAG M2 antibody (F3165, Sigma) in 300 μL phosphate-buffered saline (PBS) with 0.02% Tween 20 by agitation at room temperature for 1 h. Then, the beads were incubated with the cell lysates at room temperature for 2 h with agitation. After washed thrice with Tris-buffered saline (50 mM Tris, pH 7.4, 150 mM NaCl), the beads were resuspended in 60 μL of 2× SDS sample buffer and boiled as above to be analyzed by SDS-polyacrylamide gel electrophoresis (PAGE) and Western blotting.

### 2.8. Porcine IL-1β ELISA

Porcine IL-1β in the cell-free supernatants was determined using ELISA established in our lab previously [31]. Briefly, Immulon 2 HB U plates (#3655, Thermo Fisher) were coated with a mouse anti-porcine IL-1β antibody (MAB6811, R&D Systems) at 2 μg/mL in PBS overnight. The plates were blocked with 1% bovine serum albumin (BSA) (A7030, Sigma) in PBS for 1 h, and were incubated with either samples or the standard for 2 h. Two-fold serial dilutions of the recombinant porcine IL-1β protein (681-PI-010, R&D Systems) in diluent (0.1% BSA in Tris-buffered saline with 0.05% Tween 20) was used for the standard curve. Next, the plates were incubated with goat anti-porcine IL-1β biotinylated antibody (BAF681, R&D Systems) at 50 ng/mL in the diluent for 1 h, and further incubated for 1 h with alkaline phosphatase-streptavidin (016-050-084, Jackson ImmunoResearch, West Grove, PA, USA) that was diluted in a ratio of 1:5000 by the diluent. After incubation with 1 mg/mL of p-nitrophenyl phosphate in the diethanolamine buffer (1 M diethanolamine, 0.5 M MgCl_2_, pH 9.8), optical densities were measured at 405 nm with a reference at 490 nm using the xMark Microplate Absorbance Spectrophotometer (Bio-Rad).

### 2.9. Western Blotting

Cell lysates or IP samples were subjected to SDS-PAGE followed by blotting on nitrocellulose membranes. The membranes were blocked with 5% skim milk (or 5% BSA for the detection of phospho-DRP1) in Tris-buffered saline with 0.1% Tween 20 (TBST) for 1 h and incubated with primary antibodies in TBST at 4 °C overnight. The membranes were further incubated with secondary antibodies in TBST at room temperature for 1 h and were scanned with an Odyssey Infrared Imager (LI-COR Biosciences).

### 2.10. Confocal Microscopy

To check the mitochondrial integrity, PAMs were seeded at 2 × 10^5^ cells per well on a LabTek II CC2 chamber slide (154941, Thermo Fisher). The cells were infected with the Sk02 virus at an MOI of 1 for 7 h or they were stimulated with 200 ng/mL LPS for 16 h. After fixed with 4% paraformaldehyde for 10 min and permeabilized with 0.1% Triton X-100 in Dulbecco’s phosphate-buffered saline (DPBS) for 5 min, the cells were incubated with 5% BSA in DPBS for 1 h. The cells were probed with mouse monoclonal anti-TOM20 antibody (sc-17764, Santa Cruz, Dallas, TX, USA) and goat polyclonal anti-IAV antibody (AB1074, EMD Millipore, Etobicoke, ON, Canada) overnight at 4 °C, and the secondary antibodies (Alexa Fluor 488 donkey polyclonal anti-mouse IgG_H+L_ (A21202; Invitrogen, Mississauga, ON, Canada) and Alexa Fluor 633 donkey polyclonal anti-goat IgG_H+L_ (A21082; Invitrogen)) for 1 h at room temperature. Counterstaining was done with 4′,6-diamidino-2-phenylindole (DAPI) (D1306, Invitrogen) for 5 min and the coverslips were mounted with ProLong Diamond Antifade Mountant (P36961, Invitrogen) overnight at room temperature. Images were obtained by using a confocal laser scanning microscope (TCS SP8, Leica, Concord, ON, Canada).

### 2.11. Statistical Analysis

One-way analysis of variance (ANOVA) with Tukey’s multiple comparison tests or unpaired *t*-test with Welch’s correction was performed using GraphPad Prism 7. The error bars indicate the mean ± SD. The *p*-values of less than 0.05 were considered to be statistically significant.

## 3. Results

### 3.1. SIV Infection Induces DRP1 Phosphorylation and Mitochondrial Fission

**Figure 1:**
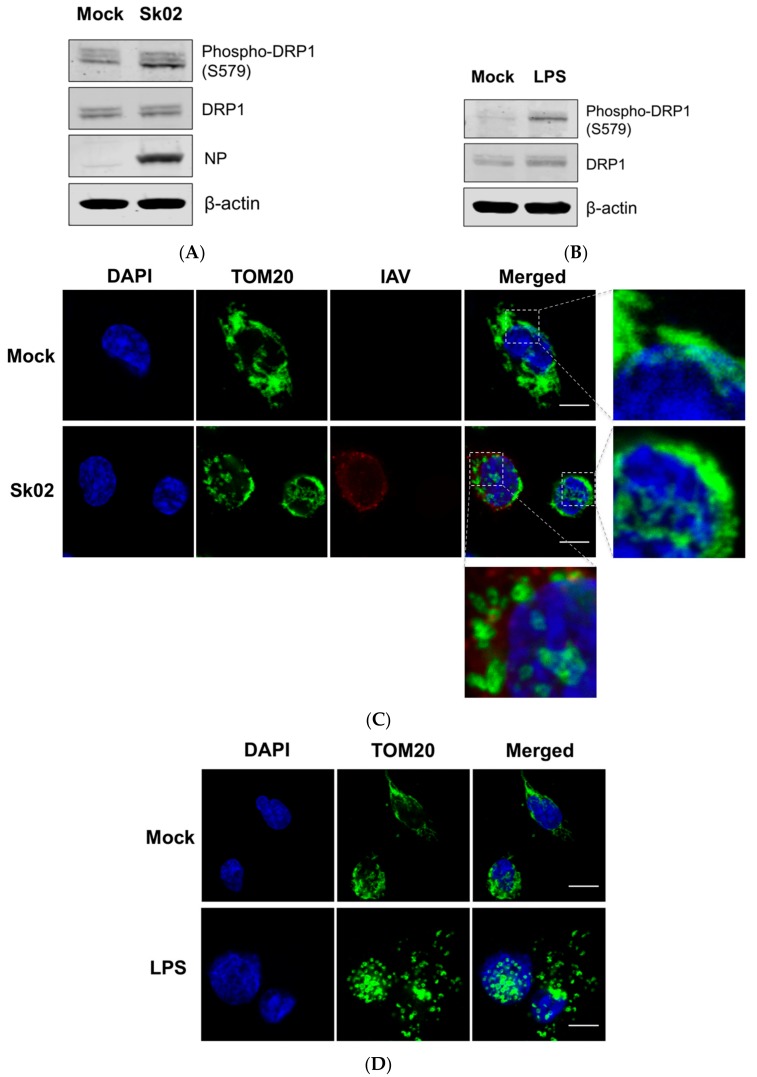
The swine influenza virus (SIV) infection induces dynamin-related protein 1 (DRP1) phosphorylation and mitochondrial fission in primary porcine alveolar macrophages (PAMs). PAMs were either infected with SIV Sk02 at an MOI of 1 for 4 h (**A**) or treated with 200 ng/mL lipopolysaccharide (LPS) for 12 h (**B**). Phospho-DRP1 at S579 and the total form of porcine DRP1 were detected by Western blotting. The viral NP protein was also monitored by Western blotting in infected cells. PAMs were either infected with Sk02 at an MOI of 1 for 7 h (**C**) or treated with 200 ng/mL LPS for 16 h (**D**). Immunofluorescence and confocal microscopy were performed using an antibody against the mitochondrial protein, TOM20, and the influenza A virus (IAV) antibody, when infected. Nuclei were stained by DAPI. Boxed areas were magnified and displayed on the right side (for a mock or un-infected cell) or below (for an infected cell) the corresponding panels in (**C**). Scale bar, 10 μm. Results are representative of three independent experiments.

It has been reported that virus-induced DRP1 phosphorylation at S616 leads to the mitochondrial fission in the early stages of viral infection [16,26,39]. Amino acid alignment indicated that S616 (location based on the transcript variant X1 of human DRP1) corresponds to S579 of porcine DRP1 (transcript variant X8) and is conserved among different isoforms of human DRP1 and porcine DRP1. We were interested in whether phosphorylation at S579 on porcine DRP1 and the subsequent mitochondrial fission would occur upon SIV infection. PAMs were infected with the Sk02 virus for 4 h at an MOI of 1 and the cell lysates were analyzed by Western blotting with antibodies specific for phospho-DRP1 (S616) or the total form of DRP1. The SIV infection indeed resulted in phosphorylation of DRP1 at S579, while the expression of total form was not changed (Figure 1A). As a positive control, PAMs were treated with LPS, which is known to induce DRP1 S616 phosphorylation or DRP1 S637 dephosphorylation-mediated mitochondrial fission in murine cells [40,41]. As expected, LPS stimulation led to DRP1 phosphorylation in the PAMs (Figure 1B).

To test whether mitochondrial fission also occurs by SIV infection, the PAMs were infected with Sk02 for 7 h at an MOI of 1. Immunofluorescence staining was conducted by probing the cells with the antibody against an outer mitochondrial membrane protein, TOM20, to observe the mitochondrial morphology. Confocal imaging revealed that in Sk02-infected cells, mitochondria exhibited short and round forms owing to fragmentation; however, in mock-infected cells, normal tubular and networked mitochondria were observed (Figure 1C). Likewise, mitochondrial fission was demonstrated in LPS-treated PAMs (Figure 1D). These data show that upon SIV infection, porcine DRP1 phosphorylation at S579 followed by mitochondrial fission occurs in PAMs.

### 3.2. SIV-Induced Porcine IL-1β Production Is Dependent on ROS Production

**Figure 2:**
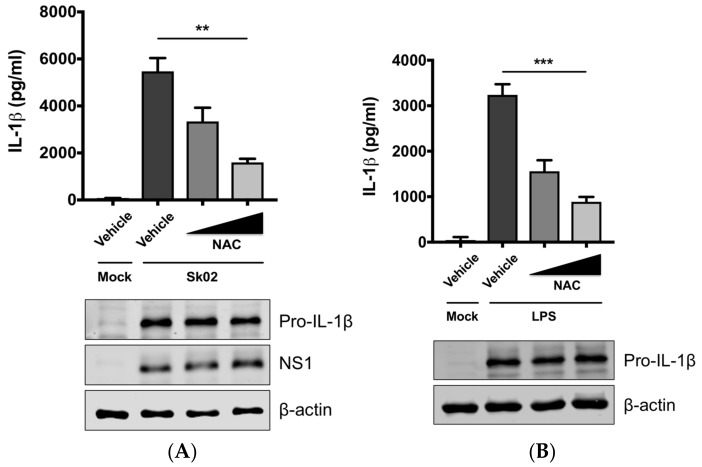
Reactive oxygen species (ROS) plays a critical role on SIV-induced IL-1β production in PAMs. PAMs were either infected with Sk02 at an MOI of 1 for 20 h (**A**) or stimulated with 200 ng/mL LPS for 16 h (**B**) in the presence of vehicle (distilled water) or increasing concentrations (2 and 10 mM) of a ROS scavenger, NAC. Porcine IL-1β levels from the supernatants were measured by ELISA and the expression of pro-IL-1β, viral NS1, and β-actin was analyzed by Western blotting. Statistical analysis was done with a one-way ANOVA. ** *p* < 0.01; *** *p* < 0.001. Results are representative of three independent experiments.

Mitochondrial ROS from damaged mitochondria can induce NLRP3 inflammasome activation [1,9] and we further examined whether IL-1β production by SIV infection is ROS-dependent. PAMs were infected with Sk02 in the presence of a ROS scavenger, NAC, or a vehicle for 20 h. In agreement with our previous report [31], the IL-1β level detected from the supernatant was considerably higher in SIV-infected cells than that in mock-infected cells (Figure 2A). Treatment of NAC significantly decreased IL-1β production in a dose-dependent manner. The expression of pro-IL-1β and the viral protein was not impaired, indicating the NAC eliminates a signal required for inflammasome activity. As a control, a decline in IL-1β levels was also achieved when the cells were treated with LPS in the presence of NAC (Figure 2B). This suggests that ROS generated from fragmented mitochondria enhances NLRP3 inflammasome-dependent IL-1β production in PAMs upon SIV infection.

### 3.3. SIV-Induced Porcine IL-1β Production Is Mediated by DRP1

**Figure 3:**
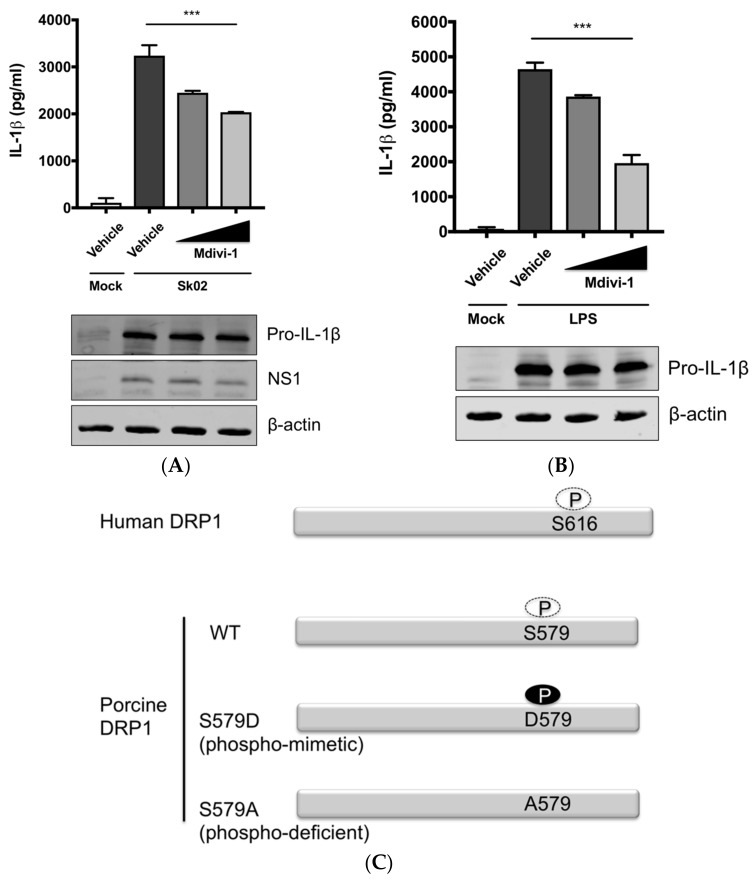

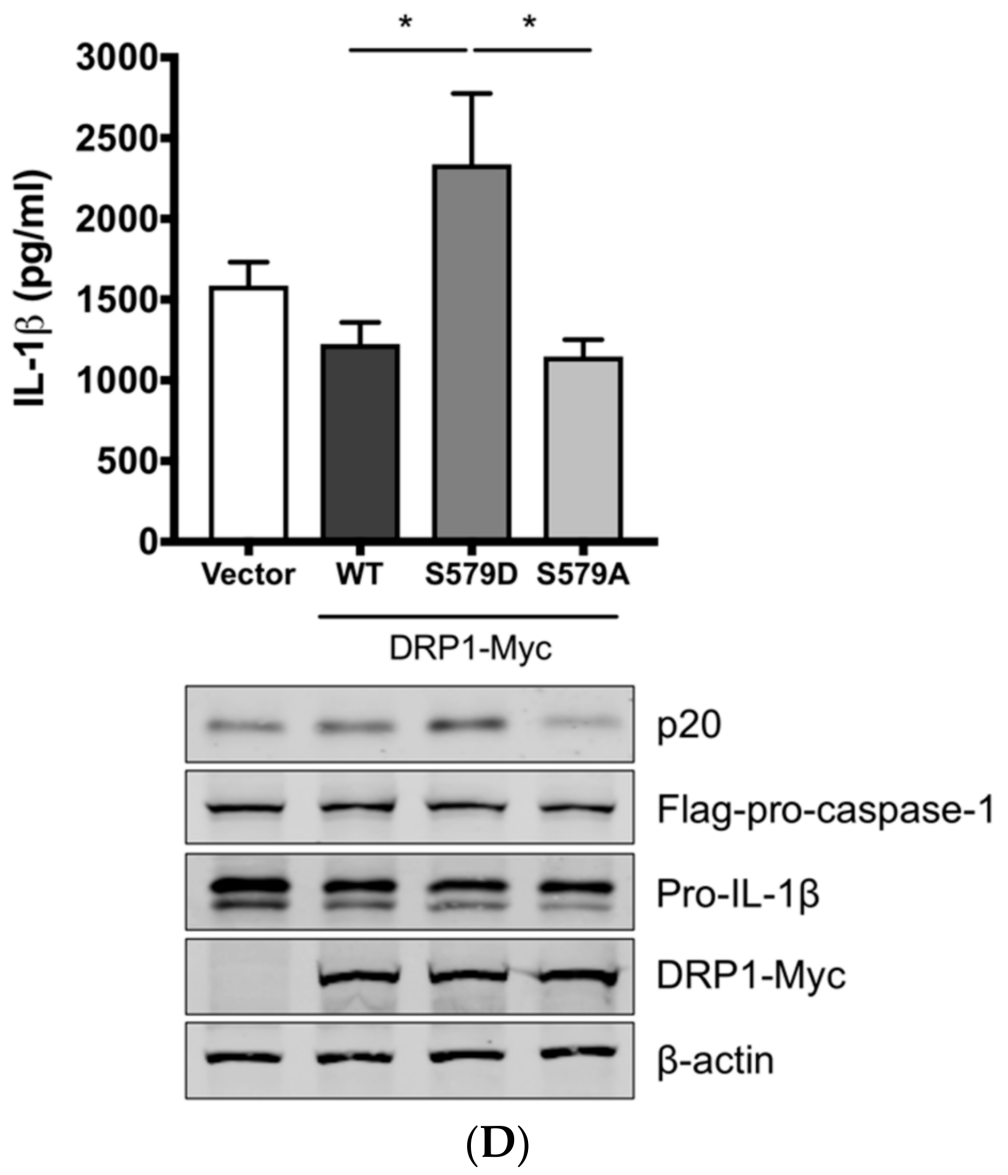
DRP1 is involved in the SIV-induced IL-1β production in PAMs. PAMs were either infected with Sk02 at an MOI of 1 for 20 h (**A**) or stimulated with 200 ng/mL LPS for 16 h (**B**) in the presence of vehicle (DMSO) or increasing concentrations (5 and 20 μM) of DRP1 inhibitor, Mdivi-1. Porcine IL-1β levels from supernatants were measured by ELISA and the expression of pro-IL-1β, viral NS1, and β-actin was analyzed by Western blotting. (**C**) Schematic representation of human DRP1 along with porcine DRP1. S579 in porcine DRP1 WT corresponds to S616 in human DRP1. D579 and A579 harbored by phospho-mimetic and phospho-deficient porcine DRP1 constructs, respectively, are displayed. (**D**) For NLRP3 inflammasome reconstitution assay, HEK293T cells were co-transfected with plasmids expressing porcine NLRP3 inflammasome components (NLRP3, ASC, and procaspase-1) and pro-IL-1β along with Myc-vector or Myc-tagged WT/mutant porcine DRP1 for 16 h. Porcine IL-1β levels from supernatants were measured by ELISA. The expression of caspase-1 (active and precursor forms) and pro-IL-1β was analyzed by Western blotting. Statistical analysis was done with one-way ANOVA. * *p* < 0.05; *** *p* < 0.001. Results are representative of three independent experiments.

The aforementioned data suggested that DRP1-mitochondrial fission axis is involved in the SIV-induced IL-1β production. To further confirm the role of DRP1, PAMs were treated with Mdivi-1, which inhibits the GTPase activity of DRP1 and mitochondrial fission [42], and were then infected with Sk02 at an MOI of 1 for 20 h. As seen before, the IL-1β level was appreciably elevated in SIV-infected cells compared to that in mock cells. The treatment with Mdivi-1 led to a decreased IL-1β production upon Sk02 infection in a dose-dependent manner. Pro-IL-1β expression and viral replication as indicated by NS1 expression were not much affected (Figure 3A). LPS-induced inflammatory cytokine expression is shown to be mediated by DRP1 activity [40], hence we also used the LPS-treated cells as a control. With the pro-IL-1β expression unaffected, the IL-1β production upon LPS was declined by the Mdivi-1 treatment (Figure 3B).

To demonstrate the role of S579-phosphorylated DRP1 on procaspase-1 cleavage, a hallmark of inflammasome activation, NLRP3 inflammasome reconstitution [31] was performed using HEK293T cells that are deficient for endogenous human NLRP3 inflammasome [43]. In this assay, the first signal required for NLRP3 inflammasome activation is provided by the overexpression of NLRP3 inflammasome components and pro-IL-1β; the regulatory effect on the second signal by the proteins and particular amino acids of interest can be examined by monitoring the levels of cleaved caspase-1 and/or mature IL-1β. Despite some limitations, as for all in vitro assays, such as excluding the roles of undefined co-factors, the system is especially useful when the cells, in this study PAMs, are difficult to be transfected. Among the different transcript variants of DRP1, we cloned porcine DRP1, which was the most identical to the variant X8 (GenBank accession number XM_021092064). S579 in porcine DRP1 corresponds to S616 in human DRP1 as explained above and as depicted in Figure 3C. Using this as the template, the phospho-mimetic (S579D) and phospho-deficient (S579A) mutants of DRP1 were generated. HEK293T cells were transfected with plasmids expressing NLRP3 inflammasome components and pro-IL-1β along with Myc-tagged vector or WT/mutant DRP1. The co-expression of phospho-mimetic DRP1 (S579D) could significantly increase the caspase-1 activity as shown by the increased level of an active form of caspase-1, p20, while not affecting the expression of pro-IL-1β (Figure 3D). In contrast, this was not observed with WT DRP1 or phospho-deficient DRP1 (S579A). Concomitantly, the porcine IL-1β level was also higher in the supernatant of cells co-expressing DRP1 (S579D) compared to other conditions. This data strongly indicate that porcine DRP1 phosphorylation at S579, which is required for mitochondrial fission, enhances the NLRP3 inflammasome activation.

### 3.4. RIPK1 Kinase Activity Is Critical for SIV-Induced IL-1β Production in PAMs

**Figure 4:**
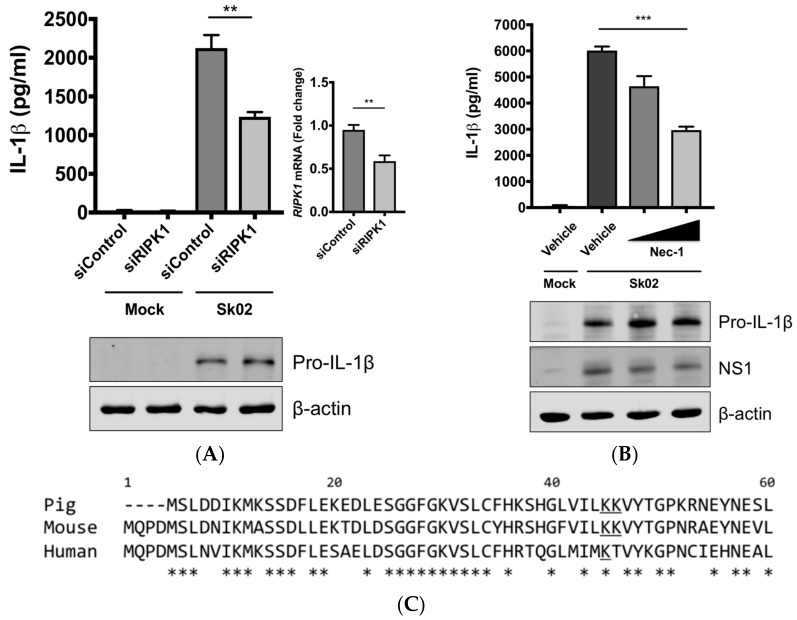

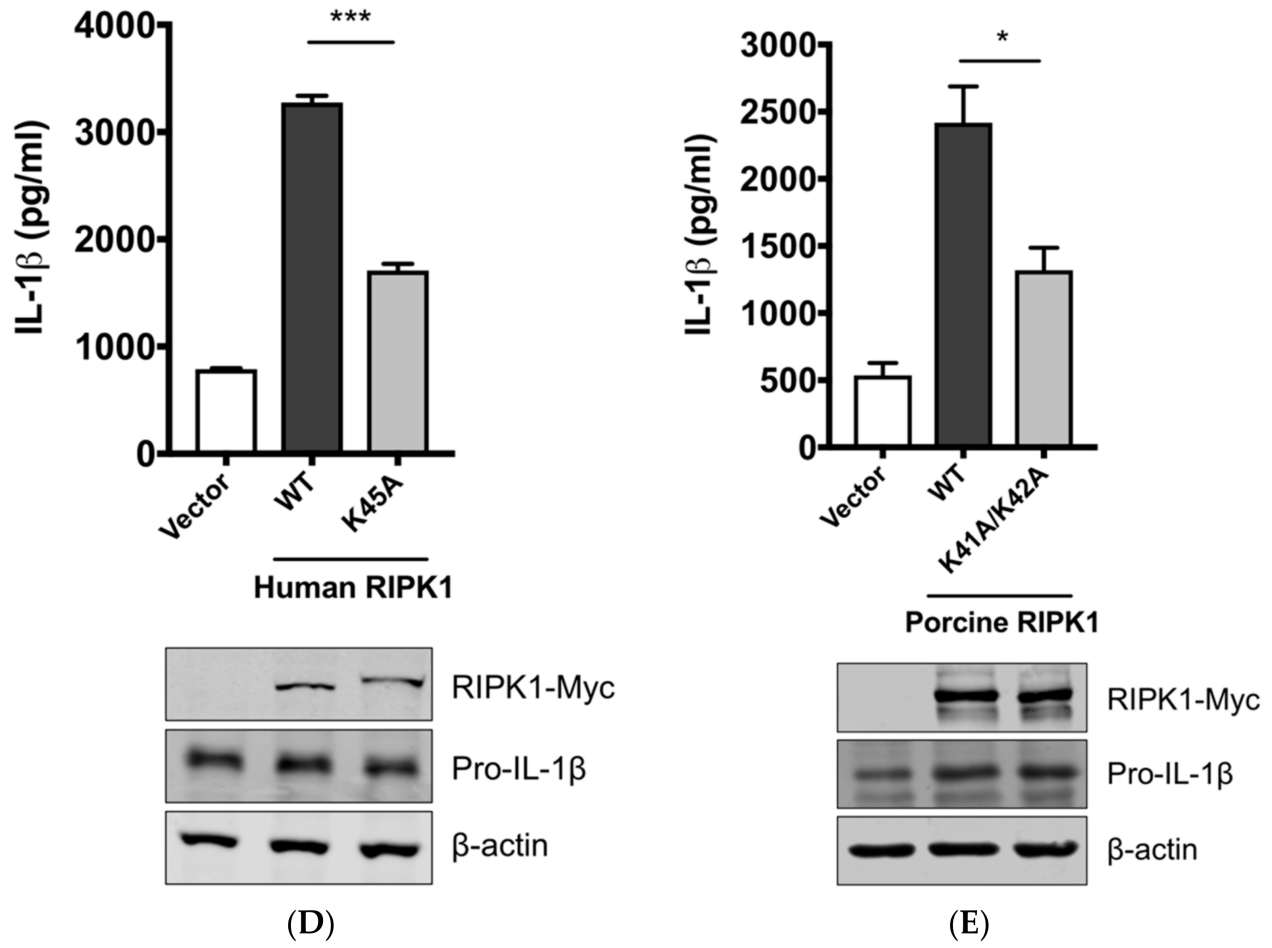
The SIV-induced IL-1β production requires receptor-interacting protein kinase 1 (RIPK1) kinase activity. (**A**) PAMs were transfected with a negative control siRNA (siControl) or *RIPK1*-targeting siRNA (siRIPK1) for 24 h and infected with Sk02 at an MOI of 1 for 16 h. Porcine IL-1β levels from supernatants were measured by ELISA. Expression of pro-IL-1β was analyzed by Western blotting. *RIPK1* knockdown efficiency was determined by real-time PCR. (**B**) PAMs were infected with Sk02 at an MOI of 1 for 20 h in the presence of a vehicle (DMSO) or increasing concentrations (80 and 160 μM) of RIPK1 kinase inhibitor, Nec-1. Porcine IL-1β levels from supernatants were measured by ELISA. Expression of pro-IL-1β and viral NS1 was analyzed by Western blotting. (**C**) Amino acid sequences of the N-terminal ends of the kinase domains in porcine, murine, and human RIPK1 (based on the GenBank accession numbers XM_005665536, NM_009068, and NM_003804, respectively) were aligned using Clustal Omega. Amino acid positions are based on the human RIPK1 and asterisks indicate conserved residues among the three species. K41/K42 in porcine RIPK1, K45/K46 in murine RIPK1, and K45 in human RIPK1 are underscored. (**D**) For the NLRP3 inflammasome reconstitution assay, HEK293T cells were co-transfected with plasmids expressing porcine NLRP3 inflammasome components (NLRP3, ASC, and procaspase-1) and pro-IL-1β along with Myc-vector or Myc-tagged WT/mutant human RIPK1 for 16 h. Porcine IL-1β levels from supernatants were measured by ELISA. Protein expression was analyzed by Western blotting. (**E**) HEK293T cells were transfected as in (**D**) along with the Myc-vector or Myc-tagged porcine RIPK1 WT/mutant for 16 h. ELISA and Western blotting were done as in (**D**). Statistical analysis was done with one-way ANOVA except for the knockdown efficiency data done with an unpaired *t*-test. * *p* < 0.05; ** *p* < 0.01; *** *p* < 0.001. Results are representative of three independent experiments.

Next, we were interested in finding the kinase that could potentially phosphorylate DRP1, leading to the activation of the NLRP3 inflammasome. We knocked down the *RIPK1* expression in PAMs by siRNA. At 24 hpt, the cells were infected with Sk02 for 16 h. IL-1β production from PAMs transfected with *RIPK1* siRNA was significantly decreased compared to the cells transfected with an off-target control siRNA, while the pro-IL-1β expression was not affected by silencing the gene (Figure 4A). The knockdown efficiency determined by the mRNA level of *RIPK1* was around 40% compared to that in negative control siRNA-transfected cells. Furthermore, to examine whether the RIPK1 kinase activity is required for upregulation of NLRP3 inflammasome activity, Nec-1, an inhibitor of the RIPK1 kinase activity [44], was used in PAMs. Nec-1 blocks the kinase function by acting on S161 on human RIPK1 [45] and this residue is conserved among different transcript variants of porcine RIPK1. Nec-1 treatment drastically reduced IL-1β production in response to the Sk02 infection in a dose-dependent manner, while not impairing the pro-IL-1β expression (Figure 4B).

Aiming to confirm the requirement of the porcine RIPK1 kinase function on IL-1β production, we aligned the amino acid sequences of the N-terminal ends of the RIPK1 kinase domain from pigs, mice, and humans (Figure 4C). Lysine 45 (K45), indicated with an underline, of human RIPK1 is a critical residue for its kinase activity [46]. While porcine and mouse RIPK1 also contain lysine at the corresponding location to K45 of their human analog, they have two consecutive lysines as underlined, K41/K42 in porcine RIPK1 and K45/K46 in mouse RIPK1. The kinase-dead effects by K45A mutation or K45A/K46T mutation on mouse RIPK1 and their roles in IL-1β production are well documented [47,48,49]. Considering the higher sequence identity of porcine RIPK1 to the human one, we first used human RIPK1 constructs in the NLRP3 inflammasome reconstitution assay. The co-expression of human RIPK1 WT remarkably increased the porcine IL-1β production. In contrast, the co-expression of kinase-dead human RIPK1 (K45A) led to a decreased level of IL-1β production compared to that of WT RIPK1 (Figure 4D). The cloning of porcine RIPK1 enabled us to conduct another experiment in the same setting as above. Consistently with human RIPK1, porcine RIPK1 WT enhanced IL-1β production, however, the kinase-dead RIPK1 (K41A/K42A) did not (Figure 4E). In all samples, the pro-IL-1β expression levels were not affected (Figure 4D,E). These suggest that RIPK1 is a positive regulator of NLRP3 inflammasome-mediated IL-1β production and its kinase activity is required for this regulation.

### 3.5. RIPK1 Interacts with DRP1

**Figure 5:**
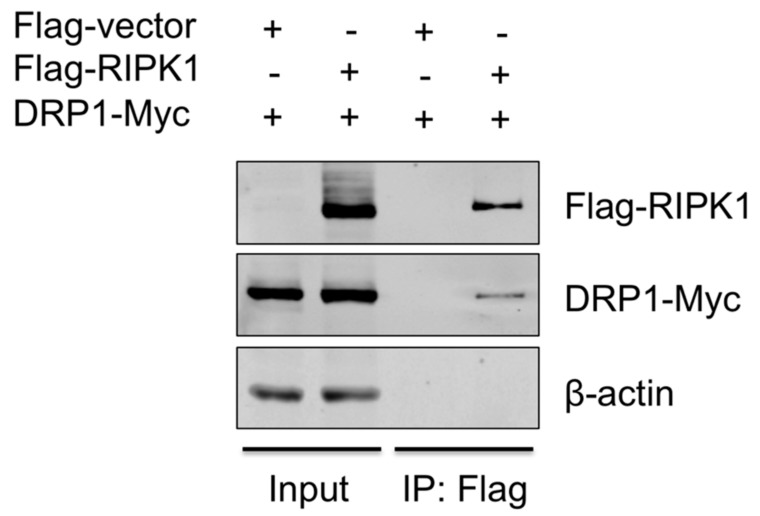
RIPK1 interacts with DRP1. HEK293T cells were co-transfected with the Flag-vector or Flag-RIPK1 construct along with the DRP1-Myc construct for 24 h. Cell lysates were subjected to co-IP with Flag antibody, and the expression of Flag-RIPK1 and DRP1-Myc in input and IP samples was analyzed by Western blotting. Results are representative of three independent experiments.

To provide the evidence that DRP1 is regulated by RIPK1, the interaction between the two proteins was examined by co-IP. Since the antibody that is against porcine RIPK1 was not available, proteins overexpressed in HEK293T, instead of endogenous proteins in PAMs, were utilized. HEK293T cells were co-transfected with Flag-vector or Flag-tagged porcine RIPK1 plasmid along with Myc-tagged porcine DRP1 plasmid for 24 h and were subjected to co-IP using the anti-Flag antibody. Association of DRP1 with RIPK1 in IP complex was demonstrated by Western blotting (Figure 5), suggesting that RIPK1 and DRP1, by physically interacting, activate the downstream events including the NLRP3 inflammasome activity.

## 4. Discussion

NLRP3 inflammasome-mediated IL-1β production is one of the key pathways in inflammatory responses to IAV infection. Alveolar macrophages, as the first responders to lung infection, play critical roles in regulating IAV-induced pulmonary inflammation [50,51,52], allowing them to be a useful model to investigate the mechanism of inflammasome activation. We previously demonstrated that the SIV Sk02 strain caused pathogenicity in pigs with typical clinical signs of SIV infection [53], and this virus induced NLRP3 inflammasome-mediated IL-1β production in PAMs [31]. Here, we explored the upstream mechanism of porcine IL-1β secretion from SIV-infected PAMs.

Changes in mitochondrial dynamics are crucial in modulating the NLRP3 inflammasome activity, and especially, ROS derived from damaged mitochondria is sensed by NLRP3 leading to the activation of NLRP3 inflammasome [9]. We speculated that mitochondrial damage and ROS production would provoke the NLRP3 inflammasome activation upon SIV infection. Indeed, in SIV-infected PAMs, mitochondrial fission and phosphorylation of DRP1, a prerequisite for its fission activity, were observed along with the ROS-dependent IL-1β production. These early events, upon SIV infection, are suggested to provide the signals required for NLRP3 inflammasome activation. Our current results and our previous finding that IL-1β levels were elevated between 8 and 16 h of SIV infection [31] support the positive regulatory role of DRP1, mitochondrial fission, and the ROS release axis in the inflammasome activation. The suppression of NLRP3 inflammasome activity by mitochondrial fission was reported in a human lung cell line in response to the mouse-adapted IAV PR8 strain during the late phase of infection [11]. Whether this discrepancy is attributed to the differences in cell types and virus strains warrants further investigation.

Our study showed that RIPK1 has critical roles in IL-1β production in PAMs. *RIPK1* silencing or inhibition of the RIPK1 kinase activity by Nec-1 sharply reduced the IL-1β production upon SIV infection. Deficiency of RIPK1 (or its kinase function) did not affect the pro-IL-1β expression, unlike mouse studies demonstrating that the RIPK1 kinase activity is required for the expression of inflammatory cytokines including IL-1β at the mRNA level [49,54,55]. In VSV-infected murine bone marrow-derived macrophages, RIPK1 promotes the NLRP3 inflammasome-mediated IL-1β production by the phosphorylation of DRP1 [16]. According to their study, cytosolic RNA sensors such as RIG-I, MDA5, DHX33, and TLR3 do not sense RNA viruses to initiate NLRP3 inflammasome activation [16]. Rather, the Z-DNA binding protein 1 (ZBP1) senses viral proteins of IAV to induce RIPK1-mediated NLRP3 inflammasome activation and cytokine production [56]. In this regard, whether ZBP1 is also an upstream sensor for SIV-induced RIPK1/DRP1 signaling to activate the porcine NLRP3 inflammasome will be an important subject for future investigation.

The present study revealed the intervening role of DRP1 phosphorylation and its fission activity in upregulating the caspase-1 activity, which is required for IL-1β production. Caspase-1 activation, in reverse, can induce mitochondrial damage, leading to pyroptotic cell death [57]. However, in our experiments, we did not notice apparent cytotoxicity by the overexpression of the phospho-mimetic DRP1 [58]. It is postulated that in addition to phosphorylation, other post-translational modifications on DRP1 [59] may occur and facilitate cell death upon virus infection. While a report describes that cell death is not mediated by RIPK1/DRP1 signaling, which is required for inflammasome activation [60], it is hard to fully exclude the possibility that cell death pathways can be involved in RIPK1/DRP1-mediated NLRP3 inflammasome activation in SIV-infected cells. Considering that RIPK1 mediates necroptosis along with inflammasome activation upon virus infection [16,56], and the inflammasome activation can lead to pyroptosis, it is possible that both cell death pathways are activated to a certain extent in SIV-infected PAMs. IAV infection induces both RIPK1/RIPK3-mediated NLRP3 inflammasome activation and RIPK3-mediated apoptosis and necroptosis [56]. We speculate that there are converging points between necroptotic and pyroptotic pathways. Further studies are required in terms of the crosstalk between the two events mediated by RIPK1. First, whether the RIPK1/RIPK3 complex is formed for necroptosis in SIV-infected cells; and second, whether RIPK1-mediated NLRP3 inflammasome activation also results in pyroptosis, if not, whether SIV interferes with the activation of gasdermin D, a caspase-1 substrate required for pyroptosis execution [61] warrant further investigation.

**Figure 6:**
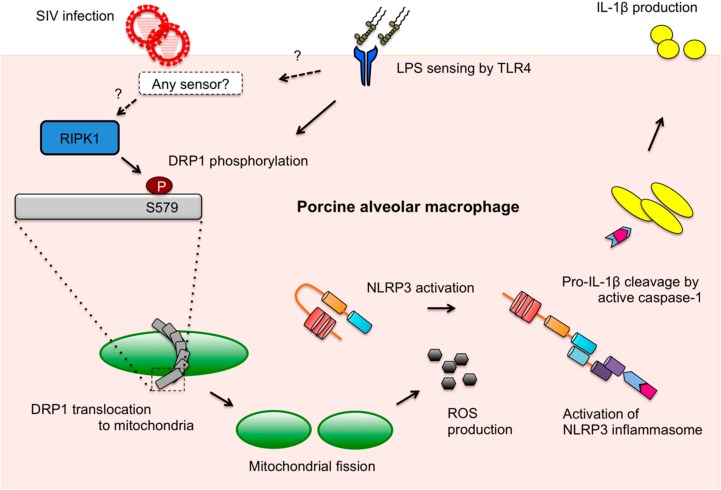
A proposed model of RIPK1/DRP1-mediated IL-1β production in SIV-infected PAMs. Recognition of IAV RNA by endosomal TLRs or RIG-I is known to induce pro-IL-1β synthesis. SIV infection turns on the RIPK1/DRP1 signaling for the NLRP3 inflammasome activation in PAMs. SIV seems to utilize the RIPK1 function, while how RIPK1 is activated by SIV or whether an upstream sensor plays a role in its activation is unclear. By interacting with DRP1 and through its kinase activity, RIPK1 induces the phosphorylation of porcine DRP1 at S579. Upon DRP1 translocation to mitochondria, mitochondrial fission occurs and ROS is generated. This promotes the activation of NLRP3 that is required for the NLRP3 inflammasome assembly and caspase-1 activation. Active caspase-1 converts pro-IL-1β into mature IL-1β, which is critical for SIV-induced lung inflammation. Sensing LPS by TLR4 is known to induce the pro-IL-1β expression, and this also induces the DRP1 phosphorylation leading to the NLRP3 inflammasome activation in PAMs, although it is unclear whether LPS activates any upstream regulator of RIPK1.

DRP1-mediated mitochondrial fission upon LPS stimulation and LPS-induced IL-1β production mediated by DRP1 activity were shown in our study. In PAMs, IL-1β production upon different ligands may be related to destructive changes in mitochondrial integrity. Although the sensing of IAV ligands and LPS upstream of the NLRP3 inflammasome activation is different between each other as illustrated in our model (Figure 6), the downstream events may partially converge on the DRP1 activation and ROS production stages for IL-1β secretion. It is notable that thioredoxin-interacting protein (TXNIP) plays as a link between ROS and NLRP3 activation. While TXNIP binds and negatively regulates the antioxidant, thioredoxin, TXNIP is released from thioredoxin on ROS production, which leads to the binding of TXNIP to NLRP3, thereby activating the NLRP3 inflammasome [62]. Hence, it is possible that this pathway is shared for IL-1β production by both SIV infection and LPS stimulation in PAMs.

Taken together, as summarized in the proposed model (Figure 6), we present that SIV induces DRP1 phosphorylation at S579 and mitochondrial fission, which leads to porcine NLRP3 inflammasome-mediated IL-1β production in PAMs. The kinase activity of RIPK1, which interacts with DRP1, is found to be critical for this process. While little is defined as to how mitochondrial dynamics fit in the innate immune response upon SIV infection, this study provides valuable information on the mechanism of SIV-induced inflammation.
